# P-1952. SARS-CoV-2 Self-Testing Modalities Demonstrate Concerning Inaccuracies Among Rural, Minority Populations: The ALL-IN SC Study

**DOI:** 10.1093/ofid/ofae631.2111

**Published:** 2025-01-29

**Authors:** Madeleine M Meyer, Stella C W Self, Virginie Daguise, Melissa Nolan

**Affiliations:** University of South Carolina, Columbia, South Carolina; University of South Carolina, Columbia, South Carolina; South Carolina Department of Health and Environmental Control, Columbia, South Carolina; University of South Carolina, Columbia, South Carolina

## Abstract

**Background:**

At-home, diagnostic self-test assays provide a unique opportunity to reduce healthcare disparities by providing low-cost, immediate access to disease testing. Despite their utility, their real-world accuracy is unknown. Therefore, this study aimed to evaluate multiple testing modalities among diverse, marginalized populations in a large, multi-year, state-wide randomized controlled trial.

**Methods:**

From 2022 to 2023, 2,195 individuals participated in testing events across South Carolina, USA. Participants provided medical personnel collected samples for gold standard laboratory comparison and were assigned to one of three groups for a matching self-test comparison. These groups were 1) no assistance self-test (only provided manufacturer self-test instructions), 2) unassisted hybrid (collected their own samples according to manufacturer instructions and mailed-in samples to laboratory), and 3) assisted (medical personnel verbally explained the manufacturer’s instructions). A robust statistical analysis plan was executed; however, only the results from multivariate regression are described here.

**Results:**

Participants in the unassisted groups were significantly more likely to have an incorrect self-test result. Conversely, participants with assistance were 32% more likely to have a correct self-test result, regardless of testing modality (saliva, dried blood spot, nasal swab). Self-collection of saliva samples to send to a laboratory for testing had the highest odds of inaccuracy (OR=9.21, p-value < 0.001). Self-collection of dried blood spot samples sent for laboratory testing had the lowest odds of inaccuracy (OR=1.52, p-value< 0.021). Demographic risk factors varied by testing modality, with females, black, and lower income participants statistically more likely to have inaccurate testing results. Select infection symptoms at the time of testing also increased the odds of inaccuracy.

**Conclusion:**

This study provides critical evidence that self-tests are a viable option for addressing health care access disparities, but telemedicine and/or community assistance programs must also be provided to ensure tests are conducted and interpreted correctly.

**Disclosures:**

All Authors: No reported disclosures

